# Global prevalence and associated factors of sleep disorders and poor sleep quality among firefighters: A systematic review and meta-analysis

**DOI:** 10.1016/j.heliyon.2023.e13250

**Published:** 2023-01-27

**Authors:** Amir Hossein Khoshakhlagh, Saleh Al Sulaie, Saeid Yazdanirad, Robin Marc Orr, Hossein Dehdarirad, Alireza Milajerdi

**Affiliations:** a Department of Occupational Health, School of Health, Kashan University of Medical Sciences, Kashan, Iran; b Department of Industrial Engineering, College of Engineering in Al-Qunfudah, Umm Al-Qura University, Makkah 21955, Saudi Arabia; c Social Determinants of Health Research Center, Shahrekord University of Medical Sciences, Shahrekord, Iran; d School of Health, Shahrekord University of Medical Sciences, Shahrekord, Iran; e Tactical Research Unit, Bond University, Gold Coast, Australia; f Medical Library & Information Sciences, School of Allied Medical Sciences, Tehran University of Medical Sciences, Tehran, Iran; g Research Center of Biochemistry and Nutrition in Metabolic Diseases, Institude for Basic Sciences, Kashan University of Medical Sciences, Kashan, Iran

**Keywords:** Global prevalence, Sleep disorders, Poor sleep quality, Firefighters, Systematic review, Meta-analysis

## Abstract

Lack of sleep can affect the health and performance of firefighters. This systematic review and meta-analysis estimated the global prevalence of sleep disorders and poor sleep quality among firefighters and reported associated factors. Four academic databases (Scopus, PubMed, Web of Science, and Embase) were systematically searched from January 1, 2000 to January 24, 2022. These databases were selected as they are known to index studies in this field. The search algorithm included two groups of keywords and all possible combinations of these words. The first group included keywords related to sleep and the second group keywords related to the firefighting profession. The relevant Joanna Briggs Institute checklist was used to evaluate study quality. Data from eligible studies were included in a meta-analysis. In total, 47 articles informed this review. The pooled prevalence of sleep disorders and poor sleep quality in firefighters were determined as 30.49% (95% CI [25.90, 35.06]) and 51.43% (95% CI [42.76, 60.10]), respectively. The results of a subgroup analysis showed that individuals in low- and middle-income countries (LMICs) had a higher prevalence of sleep disorders than those in high-income countries (HICs) but HICs had a higher prevalence of poor sleep quality than LMICs. Various factors, including shift work, mental health, injuries and pain, and body mass index were associated with sleep health. The findings of this review highlight the need for sleep health promotion programs in firefighters.

## Highlights

• The prevalence of sleep disorders in firefighters was determined as 30.49%.
• The prevalence of poor sleep quality in firefighters was determined as 51.43%.
• Individuals in low- and middle-income countries had a higher prevalence of sleep disorders.
• Individuals in high-income countries had a higher prevalence of poor sleep quality.

## 1 Introduction

Sleep disorders defined as conditions that impairs a person's sleep and prevents their restful sleep [1]. Based on the international classification of sleep disorders (ICSD), types of sleep disorders include insomnia, sleep-related breathing disorders, central disorders of hypersomnolence, circadian rhythm sleep-wake disorders, parasomnias, sleep-related movement disorders, and other sleep disorders [2]. Sleep quality is described as the person's satisfaction across all aspects of the sleep experience which in turn consists of four attributes, these being sleep efficiency, sleep latency, sleep duration, and wake after sleep onset [3]. Sleep disorders can create poor sleep quality [4].

Sleep disorders and poor sleep quality can be associated with serious impacts on an individual's physical and mental performance and productivity. Also, it can disrupt their healthy social relationships [5,6]. Research suggests that sleep deprivation weakens the immune system, decreases hypothalamus, pituitary, and adrenal function, and increases blood pressure [7]. Furthermore, Petrov et al. have highlighted the potential negative impacts of sleep disorders and sleep quality on mental health [8]. In the workplace, workers with sleep disorders have been reported to have significantly more workplace absences, which in turn, increases costs to their employer and society in general [9]. These workers are claimed to possess less self-confidence and lower job satisfaction [10]. Concerningly, research in industrial occupations suggest that sleep disorders and poor sleep quality can increase the risk of workplace accidents [11].

Firefighting is an example of an occupation where personnel work in shifts [12]. As such it is not surprising that it is one of the occupations with a high reported incidence of sleep disorders. The percentage of firefighters reporting sleep disorders ranges from 37% [13] to as high as 70% [14] with multiple percentages reported within this range [[15], [16], [17], [18], [19], [20], [21]]. Factors associated with sleep disorders in firefighters were found to include the aforementioned shift work, but also musculoskeletal disorders, higher body mass index, depression, stress, psychosomatic disorders and post-traumatic stress [13,15,[17], [18], [19], [20], [21]].

The prevalence of sleep disorders and poor sleep quality among firefighters have been found to range widely across various studies with no clear agreement on their prevalence. Furthermore, there is a sparsity of discussion regarding the factors identified as affecting sleep disorders and sleep quality. Therefore, the aims of this systematic review study and meta-analysis were to determine the global prevalence of, and the factors associated with, sleep disorders and poor sleep quality among firefighters. It was hypothesized that the prevalence of sleep disorders and poor sleep quality were higher in firefighters, particularly in those working in low-income countries.

## 2 Materials and methods

This systematic review and meta-analysis followed the Preferred Reporting Items for Systematic Reviews and Meta-Analyses (PRISMA) guidelines [22]. The methodology to be followed was reviewed and approved by the Medical Ethics Committee of Kashan University of Medical Sciences (IR.KAUMS.NUHEPM.REC.1400.044).

### 2.1 Search strategy

Searches of four academic databases (Scopus, PubMed, Web of Science, and Embase) were systematically carried out from January 1, 2000 to January 24, 2022. The search algorithm included two groups of keywords and all possible combinations of these words. The search operator of “AND” was used to combined these two groups of keywords into the search term string. The first group of keywords comprised of terms relating to sleep being. These key words included sleep problem, sleep disorder, sleep disturb, sleep debt, sleep deficien, sleep restrict, sleep depriv, sleep disrupt, sleep paralysis, sleep dysfunction, sleep quality, dyssomnia, parasomnia, restless legs syndrome, RLS, Willis Ekbom disease, periodic limb movement disorder, circadian disorders, narcolepsy, paroxysmal, narcoleptic syndrome, gelineau syndrome, hypersomnia, sleep-wake disorder, shift work sleep disorder, sleep behavior disorder, insomnia, sleep bruxism, sleep apnea, sleep loss, sleep latency, somnolence, sleep apnea syndromes, sleep wake disorders, sleep paralysis, dyssomnias, parasomnias, nocturnal myoclonus syndrome, sleep disorders, circadian rhythm, narcolepsy, disorders of excessive somnolence, sleep initiation and maintenance disorders, sleep latency, sleepiness, and sleep quality. The second group of keywords consisted of terms specific to firefighters. These keywords included firefight, fireman, fire guard, fire, fire service, and firefighter’.

### 2.2 Eligibility criteria

The following inclusion criteria were applied: a) study reported on a sleep disorder; and b) the study included firefighter populations. No filters for year of publication, age, or sex/gender, were applied. In addition, the following exclusion criteria were applied: a) the article was a review with/without meta-analysis, editorial letter, conference paper, case, or interventional or trial study; b) the article was published in a language other than English, c) the study did not report their inclusion criteria, d) the study did not report prevalence rates, or (e) the study was of poor quality based on the findings of the quality assessment.

### 2.3 Study selection

All papers identified through the searching of the various databases were entered into Endnote and duplicates were removed. Titles and abstracts of the collated papers were then screened by two reviewers (A.KH and S.Y) independently. Following this step, studies not of relevance to this review were removed. The remaining studies were then subjected to the inclusion and exclusion criteria by two reviewers (A.KH and S.Y). Studies meeting the inclusion criteria but failing to meet the exclusion criteria were retained to inform this review.

### 2.4 Quality assessment

The Joanna Briggs Institute (JBI) checklist for prevalence studies was used to evaluate the quality of the studies [23] with the evaluation performed by two researchers (A.KH and S.Y). This instrument has nine questions with four response options, including yes, no, unclear, and not applicable. The JBI tool evaluates prevalence studies based on criteria including the appropriateness of sample frame, appropriateness of sampling way, adequacy of sample size, description of study subjects and the setting, sufficiency of data analysis, the validity of used methods, reliability of measurement ways, appropriateness of statistical analysis, and adequacy of response rate. In this study, the number of positive responses were computed, and articles were categorized into three groups, including low quality (scores 1 and 2 out of 9), moderate quality (scores 3–6 out of 9), and high quality (scores 7–9).

### 2.5 Data extraction

After selecting papers, the required information was extracted by researchers. This information included first author, publication year, country, sample size, gender, study type, job type, age of participants, work experience, prevalence, sleep assessment tool, and related main factors.

### 2.6 Data analysis

The level of agreement between the two reviewers was computed using Cohen's kappa [24]. The kappa coefficients related to the first and second steps were 0.89 and 0.93, which shows a good agreement between the two reviewers. In most cases, the prevalence estimates in each study were exactly computed, but occasionally estimates were extracted from graphs or calculated using medians. Standard errors of the differences were estimated by available data whenever possible. For heterogeneity, all pooled estimates were evaluated using the Q test and I2 statistics [25]. For the primary outcome, subgroup heterogeneity and a sensitivity analysis were explored. A post hoc sensitivity analysis was also conducted. Q test and I2 statistics were applied to test for meaningful heterogeneity reduction in partitioned subgroups (country classification, study data, sample size, type of sleep assessment tools, and gender). Countries were classified into countries with low and middle income (LMIC) to high income (HIC) based on the world bank categories [26]. Study date and sample size were also divided based on the median value. Types of sleep assessment tools were categorized into four types: Type 1: The Pittsburgh sleep quality index-Epworth sleepiness scale- Epworth daytime sleepiness score; Type 2: insomnia severity index-Athens insomnia scale; Type 3: use of type 1 and type 2; Type 4: other. Genders included male and female. Publication bias was evaluated using visual inspection of the funnel plot and computation of the Begg's linear regression test [27]. Data analyses were performed using STATA 14.2.

## 3 Results

### 3.1 Study selection results and basic characteristics

**Fig. 1:**
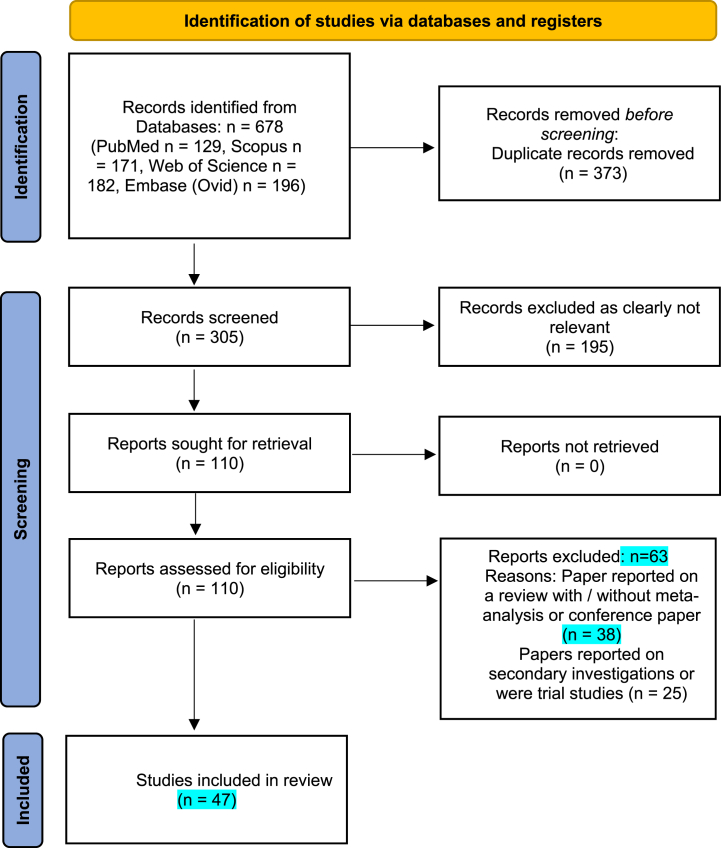
PRISMA (Preferred Reporting Items for Systematic Reviews and Meta-Analyses) flow diagram.

From the initial search, 678 papers were found. Following the removal of 373 duplicate studies, 305 studies remained to be screened by title and abstract. Finally, by eliminating 195 papers that were clearly not relevant to this study (for example ‘The sleep architecture of Australian volunteer firefighters during a multi-day simulated wildfire suppression: impact of sleep restriction and temperature’ [28]), 110 studies were submitted for consideration against the eligibility criteria. Overall, 62 papers were excluded with their reasons recorded (see Fig. 1) leaving 47 papers to inform the meta-analysis. Of these 47 papers, 34 papers reported on the prevalence of sleep disorders, 13 papers on the prevalence of poor sleep quality, and 4 papers on both sleep disorder and poor sleep quality prevalence (Fig. 1). The results from the relevant 47 studies were entered into the meta-analysis (Table 1).

**Table 1:** Characteristics of the studies included in the meta-analysis.

First author (Year)	Country	Gender	Sample size		Study Type	Job Type		Age of participants	Work experience		Sleep information	Prevalence (%)		Main factor related	Sleep assessment tool	Quality
Smith et al. (2019) [69]	United States	Male-female	652		Retrospective Cohort	Career		38.4	13.4		Sleep disorder	48.6		Alcohol misuse, distress tolerance	Pittsburgh sleep quality index	High
Angelique Savall et al. (2021) [29]	France	Male -female	193		Cross-sectional	Career/Volunteer		39.1	NR		Sleep disorder	16.1		NR	Pittsburgh sleep quality index, Epworth sleepiness scale and insomnia severity index	High
											Poor sleep quality	26.9				
Wolińska et al. (2017) [70]	Poland	Male	23		Cross-sectional	Career		33	NR		Sleep disorder	14.12		Amount of work hours, coffee drinking	Epworth sleepiness scale- Athens insomnia scale	Moderate
Víviam Vargas de Barros (2013) [21]	Brazil	Male-female	303		Cross-sectional	Career		33	10		Sleep disorder	51.2		Psychological distress, psychosomatic disturbances	General health questionnaire and sleep disturbances subscale	High
Psarros et al. (2018) [71]	Greece	Male	102		Cross-sectional	Career		40	NR		Sleep disorder	23.5		PTSD	Athens insomnia scale	Moderate
Reinberg et al. (2013) [72]	France	Male	30		Cohort	Career		37.1	17.53		Sleep disorder	60		Shift work	Self-assessments	High
Vincent et al. (2020) [73]	Australia	Male -female	60		Cross-sectional	Career		38.4	9.5		Sleep disorder	7.5		Frequency of calls	Pittsburgh sleep quality index	Moderate
Yun et al. (2015) [51]	South Korea	Female	515		Cross-sectional	Career		38.3	10.49		Sleep disorder	20.4		PTSD, stress, depressive symptoms, Chronotype	Pittsburgh sleep quality index	High
Shi et al. (2021) [74]	United States	Male -female	268		Cross-sectional	Career		42.95	19.91		Sleep disorder	42		Depression, years of professional experiences	Epworth sleepiness scale	Moderate
Wagner et al. (2018) [75]	Australia	Male -female	160		Case-control	Career		36.8	NR		Sleep disorder	10.5		NR	Pittsburgh sleep quality index	Moderate
Webber et al. (2011) [50]	United States	Male	10,342		Retrospective Cohort	Career		44.2	NR		Sleep disorder	36.5		BMI	Berlin questionnaire	High
Wróbel-Knybel et al. (2021) [76]	Poland	Male -female	831		Cross-sectional	Career		35.25	NR		Sleep disorder	8.7		PTSD, stress	Sleep paralysis experience and phenomenology questionnaire	High
Wolkow et al. (2019) [20]	United States	Male -female	6933		Cross-sectional	Career		40.4	NR		Sleep disorder	6.6		Burnout	Athens insomnia scale, Berlin questionnaire, and symptoms and treatment questionnaire	High
Khumtong and Taneepanichskul1 (2019) [18]	Thailand	Male	1215		Cross-sectional	Career		39.2	11.5		Poor sleep quality	49.1		PTSD	Pittsburgh sleep quality index	High
Korre et al. (2016) [77]	United States	Male	400		Cross-sectional	Career		47	NR		Sleep disorder	20.86		BMI	Berlin questionnaire	High
Kim and Ahn (2021) [78]	South Korea	Male	297		Case-control	Career		38.9	NR		Sleep disorder	41.75		NR	Insomnia severity index	High
Park et al. (2019) [79]	South Korea	Male	287		Cross-sectional	Career		40.1	NR		Sleep disorder	31.1		NR	Athens insomnia scale	High
Hom et al. (2017) [80]	United States	Male -female	929		Cross-sectional	Career		38.93	12.6		Sleep disorder	51.9		NR	Insomnia severity index	High
Abbasi et al. (2020) [60]	Iran	Male	118		Cross-sectional	Career		33.30	8.35		Sleep disorder	50.8		Musculoskeletal disorders	Insomnia severity index	High
Angehrn et al. (2020) [49]	Canada	Male - female	760		Cross-sectional	career/volunteer		18–64 (range)	NR		Sleep disorder	49		PTSD, depression, anxiety, social anxiety disorder, panic disorder, alcohol use disorder	Insomnia severity index	High
Barger et al. (2015) [16]	United states	Male - female	6933		Cross-sectional	Career		40.40	13.7		Sleep disorder	37.2		NR	Sleep disorders screening questionnaires	High
Sullivan et al. (2017) [81]	United states	Male - female	431		Cohort	Career		42.70	15.65		Sleep disorder	41.5		NR	Sleep disorders screening questionnaires	High
Haddock et al. (2013) [81]	United states	Male	458		Cohort	Career		38.2	13.8		Sleep disorder	18.1		48-h work shifts, non-private department sleep areas, working a second job outside the fire service	Epworth sleepiness score	High
Hom et al. (2016) [17]	United states	Male-female	880		Cross-sectional	Career/volunteer		18 to 82 (range)	NR		Sleep disorder	52.7		NR	Insomnia severity index	High
Jang et al. (2020) [44]	South Korea	Male-female	9738		Cross-sectional	Career		<40 to >50	NR		Sleep disorder	41.8		Type of job, the frequency of emergency and off-duty work	Insomnia severity index	High
Kim et al. (2021) [82]	South Korea	Male-female	51149		Cross-sectional	Career		40.70	11.70		Sleep disorder	16.1		PTSD, AUDs, depression	Berlin questionnaire	High
Kwak et al. (2020) [45]	South Korea	Male-female	352		Prospective before–after	Career		40.1	NR		Sleep disorder	47.16		NR	Insomnia severity index	High
Lim et al. (2020) [83]	South Korea	Male-female	325		Cross-sectional	Career		41.13	NR		Sleep disorder	8.48		Shift work	Pittsburgh sleep quality index, insomnia severity index and Epworth sleepiness scale	High
											Poor sleep quality	50.15				
Lim et al. (2020) [84]	South Korea	Male-female	602		Cross-sectional	Career		30.72	NR		Sleep disorder	4		Caffeine intake, shift work, circadian rhythm type, depression, anxiety, stress and social support	Pittsburgh sleep quality index and insomnia severity index	High
											Poor sleep quality	42.4				
Lim et al. (2020) [85]	South Korea	Male-female	9788		Cross-sectional	Career		39.58	NR		Sleep disorder	9.1		levels of feeling of job loading, levels of physical strength utilization rate, frequency levels of occupational activities, high-intensity leisure-time physical activities	Insomnia severity index	High
Lusa et al. (2002) [65]	Finland	Male	543	Cross-sectional			Career	<35 to >54	NR	Sleep disorder			61	Duration of working, alcohol consumption, smoking	Three-levels variable based on two questions	High
Lusa et al. (2015) [62]	Finland	Male	360	Cohort			Career	35.7	NR	Sleep disorder			41.94	NR	A self-administered questionnaire (two questions)	High
Choi et al. (2020) [86]	South Korea	Male-female	60	Cross-sectional			Career	42.80	NR	Sleep disorder			31.65	Depressive mood and anxiety symptoms	Insomnia severity index and Epworth sleepiness scale	High
Cramm et al. (2021) [87]	Canada	Male-female	1217	Cross-sectional			Career/volunteer	18 to 60 <	>4 to 16 <	Sleep disorder			21.3	NR	Insomnia severity index and five-point scale of sleep quality	High
										Poor sleep quality			69.2			
Vasconcelos et al. (2021) [88]	Brazil	Male-female	493	Cohort			Career	24.3	NR	Sleep disorder			22.1	NR	Single question on sleep problems	Moderate
Stout et al. (2021) [89]	United States	Male -female	45	Cross-sectional			Career	37.4	NR	Poor sleep quality			83.95	Shift work	Pittsburgh sleep quality index and actigraphy	High
Mehrdad et al. (2013) [14]	Iran	Male	379	Cross-sectional			Career	33.01	9.74	Poor sleep quality			69.9	Having another job, smoking, years of job experience	Pittsburgh sleep quality index	High
Hernandez et al. (2017) [90]	United States	Male	79	Cross-sectional			Career	NR	NR	Poor sleep quality			59.5	NR	Pittsburg sleep quality index	High
Demiralp and Özel (2021) [53]	Turkey	Male	43	Cross-sectional			Career	NR	NR	Poor sleep quality			8	Shift work	Pittsburgh sleep quality index	High
Abbasi et al. (2018) [15]	Iran	Male	118	Cross sectional study			Career	33.30	8.35	Poor sleep quality			59.3	Musculoskeletal disorders, shift work, BMI, job stress	Pittsburgh sleep quality index	Moderate
Oh et al. (2018) [91]	South Korea	Male - female	120	Cross-sectional			Career	38.00	NR	Poor sleep quality			52.69	Alcohol consumption, depression, anxiety, experience of traumatic events	Pittsburgh sleep quality index	Moderate
Billings et al. (2016) [43]	United states	Male	109	Cross-sectional			Career	38.0	12.70	Poor sleep quality			73.4	Shift work, personal average number of night interruptions at work, working a second job	Pittsburgh sleep quality index	High
Carey et al. (2011) [92]	United states	Male-female	112	Cross-sectional			Career	43.6	15.5	Poor sleep quality			59	Depression, physical/mental well-being, drinking behaviors (hazardous drinking and caffeine overuse), and age.	Pittsburgh sleep quality index and Epworth sleepiness scale	High
Jeong et al. (2019) [93]	South Korea	Male-female	359	Cross-sectional			Career	<40 to >50	NR	Poor sleep quality			55.4 (Control group) 72.8% (day work) 92.9% (night work) 78.2% (rest day)	Shift work	Actigraphy and sleep diary	High
Kim et al. (2020) [94]	South Korea	Male-female	1022	Cohort			Career	41.77	NR	Poor sleep quality			52.45	NR	Pittsburgh sleep quality index	High
Lim et al. (2014) [13]	South Korea	Male	657	Cross-sectional			Career	<29 to >50	<10 to >20	Poor sleep quality			48.7	Shift work, musculoskeletal disorders, depression	Pittsburg sleep quality index	High
Marconato and Monteiro (2015) [95]	Brazil	Male	71	Cross-sectional			Career	36.4	NR	Poor sleep quality			17.8	NR	–	Moderate

NR = Not Reported; PTSD: post-traumatic stress disorder; AUDs: alcohol use disorders; BMI: body mass index.

In total, 28 studies included both male and female participants, while 19 studies included only male participants, and one study only female participants. The majority of the research was carried out in the USA (n = 15 studies) and in South Korea (n = 14 studies). Other countries included Brazil and Iran (n = 3 studies respectively), Australia, Canada, Finland, France, and Poland (n = 2 studies respectively), and Greece, Thailand, and Turkey (n = 1 study respectively).

### 3.2 Quality assessment

The JBI checklist was used to assess the quality of articles. The results indicated that almost 80% of the included studies were of a high quality, being scores of 7/9 or higher. This suggests that the volume of evidence presented in this review was of high quality.

### 3.3 Global prevalence of sleep disorders in firefighters

#### 3.3.1 Results of meta-analysis

**Fig. 2:**
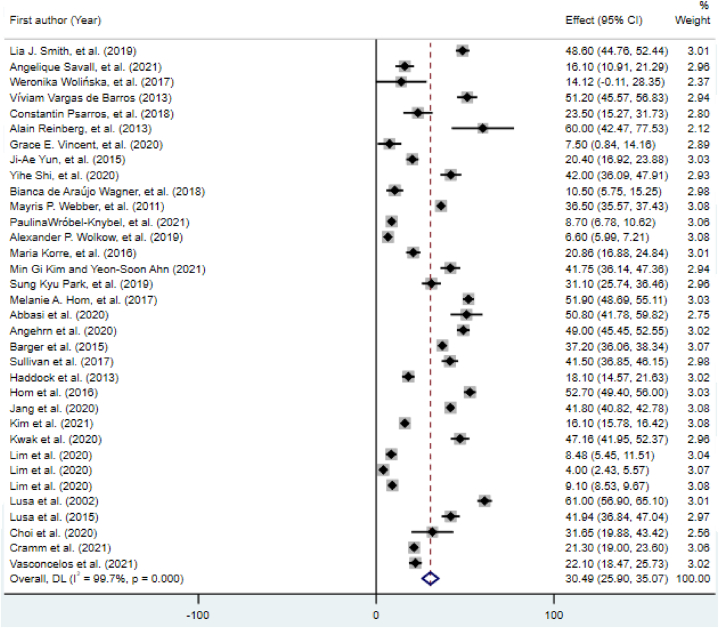
Forest plot of the prevalence of sleep disorders.

**Fig. 3:**
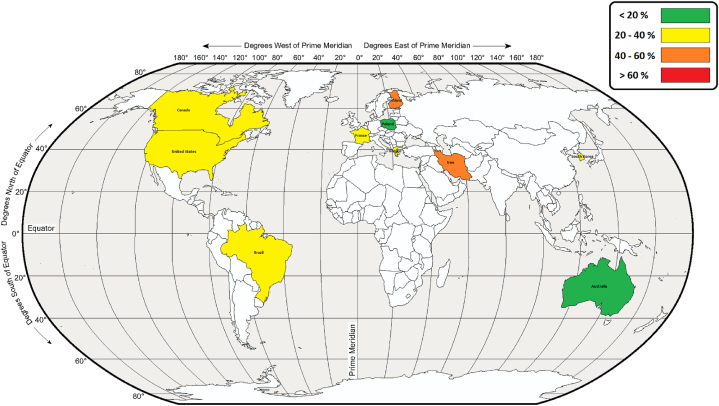
The global map of prevalence of sleep disorders in various countries.

Based on the meta-analysis results, the pooled prevalence of sleep disorders in firefighters was 30.49% (95% CI [25.90, 35.06]; Fig. 2). Fig. 3 shows the global map of prevalence of sleep disorders in various countries. Computation of the Egger's test (t = 2.47, 95% CI [1.53, 16.03], p = 0.019) identified a publication bias.

#### 3.3.2 Results of subgroup analyses

The findings of the subgroup analyses are presented in Table 2. Individuals in LMICs had a higher prevalence of sleep disorders than those in HICs (41.17%, 95% CI [19.15, 63.19] vs. 29.48%, 95% CI [24.70, 34.25]). A significant heterogeneity was seen among the studies, according to the Q test (p < 0.001) and I2 statistics. The sleep disorder prevalence in firefighters is shown in Fig. 2 as a random effects model where the grey box shows the prevalence and the length of the line at which the grey box lies the 95% confidence interval for each study. The diamond sign shows the pooled prevalence globally for all studies.

**Table 2:** Subgroup analysis and univariate meta-regression results for the prevalence of sleep disorders.

Subgroups	Subgroup analysis					
	Categories (No. of studies)	Pooled prevalence percentage (95% CIs)	I2 (%)	Q statistic (d.f.)	P-value of heterogeneity	Begg's test z (p)
Country classification (income level)	HICs (n = 31)	29.48 (24.70–34.25)	99.7	10052.4 (30)	<0.001	0.07 (0.946)
	LMICs (n = 3)	41.17 (19.15–63.19)	97.8	89.67 (2)	<0.001	0.0 (1)
Study date	Before median of 2019 (n = 16)	36.79 (31.57–42.02)	98.1	776.38 (15)	<0.001	1.04 (0.3)
	Equals median or later (n = 17)	26.16 (20.90–31.41)	99.7	4927.15 (16)	<0.001	0.75 (0.450)
Sample size	Below median: n < 352 (n = 13)	29.50 (19.84–39.16)	96.9	387.5 (12)	<0.001	1.04 (0.3)
	Median or greater: n = ≥352 (n = 21)	31.15 (25.44–36.87)	99.8	9814.7 (20)	<0.001	0.42 (0.673)
Gender	Female (n = 1)	20.40 (16.92–23.88)	N/A	N/A	N/A	N/A
	Male (n = 11)	36.02 (28.48–43.55)	97.1	346.22 (10)	<0.001	0.31 (0.755)
	Male- female (n = 22)	28.31 (23.00–33.63)	99.7	7850.93 (21)	<0.001	0.39 (0.693)
Type of sleep assessment tool	Type 1 (7)	21.94 (9.507–34.374)	99.1	654.00 (6)	0.001	0.15 (0.881)
	Type 2 (11)	38.166 (25.110–51.223)	99.8	4545.24 (10)	<0.001	−1.17 (0.243)
	Type 3 (5)	12.971 (6.513–19.428)	90.7	42.90 (4)	<0.001	0.98 (0.327)
	Type 4 (11)	35.395 (26.729–44.061)	99.7	3474.99 (10)	<0.001	0.23 (0.815)

LMIC = low- and middle-income countries: HIC = high-income countries.

#### 3.3.3 Results of meta-regression

To study the effects of potential parameters on the heterogeneity of sleep disorder prevalence in firefighters, meta-regression was applied for age, the sample size, and year of survey. Univariate meta-regression analysis revealed that mean age (slope = 0.32, p = 0.725), sample size (slope = −0.0001, p = 0.713), and survey year (slope = −1.90, p = 0.153) were not associated with sleep disorder prevalence. According to findings, with the increase in the age of participants the prevalence of sleep disorder in firefighters increases, although not significantly (p = 0.725). Similarly, findings showed that with the increase in the sample size the prevalence of sleep disorder in the firefighters also increased, again not significantly (p = 0.713). Furthermore, with the furthering of the years of the surveys, the prevalence of sleep disorder in firefighters also increased, again not significantly (p = 0.153).

### 3.4 Global prevalence of poor sleep quality in firefighters

#### 3.4.1 Results of meta-analysis

**Fig. 4:**
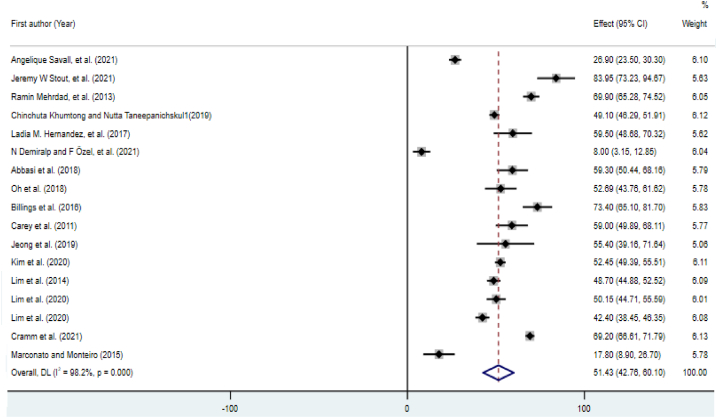
Forest plot of the prevalence of poor sleep quality.

**Fig. 5:**
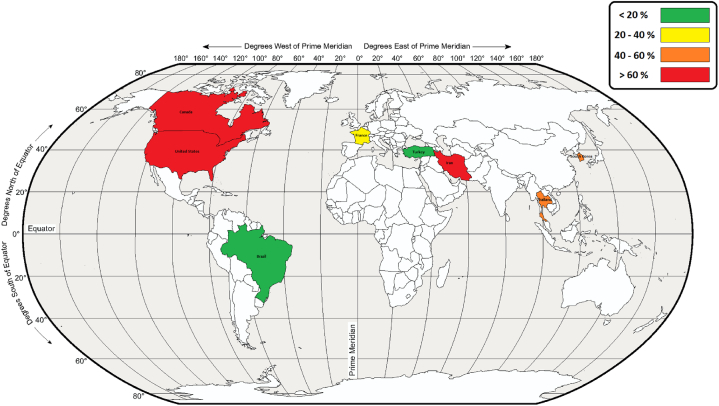
The global map of prevalence of poor sleep quality in various countries.

According to the data from 18 studies, the pooled prevalence of poor sleep quality was 51.43% (95% CI [42.76, 60.10]; Fig. 4). Fig. 5 shows the global prevalence of poor sleep quality in various countries. The Begg's test (z = 0.54, p = 0.592) did not identify a publication bias.

#### 3.4.2 Results of subgroup analyses

In the subgroup analyses (Table 3), HICs had a higher prevalence of poor sleep quality than LMICs (55.83%, 95% CI [46.61–65.04] vs. 40.85% 95% CI [18.52, 63.19]). A significant heterogeneity was seen among the studies, according to Q test (p < 0.001) and I2 statistics.

**Table 3:** Subgroup analysis and meta-regression results for the prevalence of poor sleep quality.

Subgroups	Subgroup analysis					
	Categories (No. of studies)	Pooled prevalence percentage (95% CIs)	I2 (%)	Q statistic (d.f.)	P-value of heterogeneity	Begg's test z (p)
Country classification (income level)	HICs (n = 12)	55.83 (46.61–65.04)	97.6	467.36 (11)	<0.001	1.03 (0.304)
	LMICs (n = 5)	40.85 (18.52–63.19)	99.0	388.93 (4)	<0.001	0.73 (0.462)
Study date	Before median of 2019 (n = 7)	52.49 (40.89–64.09)	95.0	119.97 (6)	<0.001	0.00 (1)
	Equals median or later (n = 10)	50.75 (38.69–62.82)	98.8	759.42 (9)	<0.001	0.18 (0.858)
Sample size	Below median: n < 352 (n = 11)	49.38 (35.22–63.54)	97.7	438.97 (10)	<0.001	−0.54 (0.586)
	Median or greater: n = ≥352 (n = 6)	55.28 (46.23–64.33)	97.7	222.21 (5)	<0.001	−1.32 (0.188)
Gender	Male (n = 8)	48.13 (33.43–62.84)	98.4	438.09 (7)	<0.001	0.12 (0.902)
	Male- female (n = 9)	54.38 (42.85–65.92)	98.2	437.45 (8)	<0.001	0.73 (0.466)
Type of sleep assessment tool	Type 1 (12)	52.608 (42.288–62.929)	97.8	492.51 (11)	<0.001	0.41 (0.681)
	Type 2 (3)	46.186 (19.455–72.918)	99.5	399.88 (2)	<0.001	−0.52 (0.602)
	Type 3 (1)	50.150 (44.714–55.586)	N/A	N/A	N/A	N/A
	Type 4 (1)	51.429 (42.762–60.095)	N/A	N/A	N/A	N/A

LMIC = low- and middle-income countries: HIC = high-income countries.

#### 3.4.3 Results of meta-regression

Univariate meta-regression analysis showed that mean age (slope = −0.657, p = 0.671), sample size (slope = −0.004, p = 0.172), and survey year (slope = 0.198, p = 0.922) were not associated with the percentage of poor sleepers.

#### 3.4.4 Factors affecting sleep disorders and poor sleep quality in firefighters

To add further context to this paper, the main determinants of sleep disorders and poor sleep quality were reviewed systematically. Some papers (16 papers) investigated the association between sleep disorders/poor sleep quality and medical condition, such as musculoskeletal disorders or mental health, response to traumatic events, distress, and depression. Other papers (17 papers) investigated the association between sleep disorders/poor sleep quality and chronotype, shift work, and other career aspects. The evidence suggests that shift work may be associated with sleep problems and poor sleep quality in firefighters, especially if firefighters are working “out-of-phase” with regard to chronotype.

## 4 Discussion

Of the initial 678 papers identified in this systematic review, 47 studies from 12 countries spanning 5 continents (North America, South America, Asia, Australia/Oceania, and Europe) were retained. All 47 studies were included in the final meta-analysis, with 34 studies presenting on the prevalence of sleep disorders and 13 studies on the prevalence of sleep quality (4 studies presenting on both sleep disorder and poor sleep quality prevalence). This systematic review and meta-analysis is the first known review that presents a quantitative estimation of subjective sleep disorders and poor sleep quality among firefighters globally.

Sleep disorders are common but stay mainly undiagnosed and untreated in the general population, as well as in firefighters [16,29]. Furthermore, sleep disorders among firefighters are considered to be greater than that found in the general population [21,30]. However, the quantitative synthesis performed in the current study shows that the global pooled prevalence of sleep disorders was 30.49% (95% CI [25.90, 35.06]). These data are lower than those found in some populations but higher than those in others. In health care personnel. For example, the percentage of sleep disturbances in Italian nurses facing COVID-19 has been reported at 71.4% [31] and in Pakistani nurses at 74.9% [32]. Conversely, the percentage of sleep disorders among female and male Egyptian public officials were found to be lower at 26.2% and 14.5% [33]. Similar to the results of this study, a comprehensive study by Barger et al. (2015) about sleep disorder screening program in firefighters found that 37.2% of firefighters reported symptoms in accordance with at least one sleep disorder. In that study the most prevalent sleep disorders were obstructive sleep apnea (OSA), followed by shift work disorder, insomnia, and restless legs syndrome [16].

Considering the above findings of differences in prevalence, the pooled prevalence of sleep disorders in this review was found to be considerably higher in LMICs (41.17%) than in HICs (29.48%). An explanation for these results can follow that key cultural, demographic, geographical, and health factors may tend towards lower population risk in HICs [34]. This supposition is supported by previous global reviews which found higher rates of sleep disorders in LMICs [[34], [35], [36]]. Support for the findings of this review can be drawn from a cross-sectional survey conducted of 16,680 residents who were 65 years old or older in catchment areas of Cuba, the Dominican Republic, Peru, Venezuela, Mexico, China, India, and Puerto Rico. The prevalence of sleep complaints in that study ranged from 9.1% (China) to 37.7% (India) [35]. The higher rates of sleep disorders in LMICs can be due to a number of reasons ranging from the number firefighter facilities to the number of firefighter personnel and the number of fire incidents attended. In HICs, the firefighting facilities and available equipment are present in greater numbers than in LMICs, reducing the number of fires attended by individual firefighters [37]. This exposure to fire incidence attendance is further reduced by the generally higher number of personnel in HIC. As such, the higher pooled prevalence in LMICs highlights the importance of considering sleep disorders as a public health concern, especially in LMICs who already face challenges imparted by communicable and nutritional deficiency diseases. These LMIC impacts are seen in other occupations as well. For example, a scoping review of healthcare employees during the COVID-19 pandemic in LMICs reported that insomnia and poor sleep quality were common in healthcare employees [38]. The differences in prevalence between income country categories, highlights the importance of considering the research in the context of the countries the findings were drawn from and highlights the strengths of this review in which studies were drawn from 5 continents.

In regard to sleep quality, the data reported in this review identified that firefighters presented with a concerning level of poor sleep quality with more than half (51.43%) making a complaint in this regard. This reported prevalence of poor sleep quality is higher than the prevalence observed in frontline health professionals (18.4%) [39] and in workers who came back to work during the COVID-19 pandemic (14.9%) [40], but lower than that reported in the nursing staff (61%) [41]. Furthermore, in contrast to the prevalence findings for sleep disorders, the pooled prevalence of poor sleep quality was largely higher in HICs (55.83%) than in LMICs (40.85%). These findings may have been due to several factors such as higher levels of substance use, psychological stress, and access to and consumption of caffeinated beverages, all of which can affect sleep quality [34,42].

Several studies [[43], [44], [45]] have reported that work seniority was correlated with higher sleep quality, the results indicating that adaptive mechanisms and strategies might intervene. However, shift work was found to lead to poorer sleep outcomes [[43], [44], [45]]. Polysomnographic surveys have shown the existence of a relationship of dose-response between shift work duration and the frequency of changing sleep patterns [46,47]. These findings are supported by the work of Lim et al. [13] who identified a significant relationship between sleep disorders and musculoskeletal disorders, depression, and shift work. As such, the evidence in relation to sleep quality and seniority and shift work is of note given that firefighters present as a population at increased risk of sleep disorders. These findings inform the importance of, and potential approaches to, sleep health promotion programs in firefighters. These programs can be included in already successful healthy lifestyle studies being conducted with firefighters [48] and can be especially helpful for firefighters at the beginning of their occupation and for those on evening/night shift.

Chronic exposure to routine stressors encountered in the firefighters’ general work environment was observed to be largely associated with poor sleep quality and sleep disorders [20,49,50]. This chronicity is of concern, given associations between poor sleep quality and mental health. For example, De Barros et al. in a study on 303 firefighters, reported that sleep disturbances were significantly associated with psychological distress and psychosomatic disturbances [21]. Likewise, Khumtong et al. found that poor sleep quality were associated with post-traumatic stress symptoms (PTSD) [18]. This report, in agreement with findings presented by other authors [15,51], suggests that stress associated with routine and ongoing operations can be more detrimental to the health of the firefighters than exposure to a single stressful event: A finding that may be of benefit to health promotion program content.

There are some data indicating that good sleep quality can protect the human body from a variety of metabolic and nutritional disorders [52]. Demiralp and Özel reported that poor sleepers in Turkish firefighters presented with a higher prevalence of metabolic syndrome (MetS) than mine industry workers [53]. Research confirms that sleep deprivation has a causal association with weight gain. Insomnia, for example, can lead to obesity through fatigue and subsequent decreased physical activity [54]. At a higher order level, brain activity (frontal cortex) has been noted to increase in response to food stimuli in people with chronic sleep deprivation [55]. Thus, it is not surprising that higher BMIs are associated with poorer sleep quality among firefighters [15,29,[56], [57], [58]], and, given that BMI is associated with MetS, that MetS prevalence in firefighters with poor sleep quality is high [53].

Musculoskeletal disorders (MSDs) are a notable concern for firefighters [59]. As such, findings that report connections between MSDs and sleep disorders in firefighters are of note [15,[60], [61], [62]]. While the associations between MSDs and sleep disorders were only found in relation to sleep quality and low back pain, sleep disorders can worsen pain and inflammatory processes through a reduction in endogenous pain inhibitors which occurs as insomnia severity increases [63]. Hence the findings by Salazar et al. [64] who reported that people with pain-related sleep disorders are remarkably more disabled. Considering the findings of relationships between MSDs, pain and sleep, there is also a complicated mutual relationship between mental health parameters (stress and depression) and occupational MSDs [15,65]. Research by Kim et al. [66], as an example, found that both occupational stress and MSDs were prevalent in firefighters. The co-existence of these factors, being MSDs, pain, occupational stress and depression, and sleep pattern can act in synergy to impact on firefighter wellness. Therefore, health and wellbeing interventions that target any of these factors may be of benefit to firefighters sleep quality and improve life quality [67,68]. As a limitation to this review, most of the studies were conducted in developed countries whereas sleep disorders and associated concerns may be greater in developing countries with low or medium incomes, Africa in particularly. Further limitations include the limited number of cohort studies compared to cross sectional studies.

## 5 Conclusion

The findings of this systematic review with meta-analysis, which consisted of generally high quality (80%+) research papers from across 5 continents, identified that the global prevalence of poor sleep quality in firefighters was high with sleep disorders considerably higher in LMICs than in HICs but poor sleep quality considerably higher in HICs than in LMICs. Sleep disorders and poor sleep quality are a notable and relevant concern among firefighters and can cause considerable damage to both their health and physical wellbeing. As such, it is recommended that sleep health promotion programs are provided to firefighters and should be implemented at the beginning of their occupation and reinforced for those on evening/night shift. Likewise, reducing MSDs, pain management and optimizing mental health may feed into, and be an outcome of, sleep health promotion programs. Thus, the volume of evidence in this review suggests that the sleep health (and subsequent health and wellbeing) of firefighters should be addressed from both a public and occupational health perspective (e.g., BMI, mental health, shift work planning, etc.) and an occupational medicine perspective (e.g., reducing MSDs and pain).

## Ethics approval and consent to participate

The methodology to be followed was reviewed and approved by the Medical Ethics Committee of Kashan University of Medical Sciences (IR.KAUMS.NUHEPM.REC.1400.044).

## Consent for publication

Not applicable.

## Availability of data and materials

The datasets used and/or analyzed during the current study are available from the corresponding author on reasonable request.

## Author contribution statement

All authors listed have significantly contributed to the development and the writing of this article.

## Funding statement

Amir Hossein Khoshakhlagh was supported by Kashan University of Medical Sciences [IR.KAUMS.NUHEPM.REC.1400.044].

## Data availability statement

Data will be made available on request.

## Declaration of interest’s statement

The authors declare no conflict of interest.
