# Supplementary material for: Global prevalence and associated factors of sleep disorders and poor sleep quality among firefighters: A systematic review and meta-analysis
Source: Heliyon. 2023 Jan 27;9(2):e13250. doi: 10.1016/j.heliyon.2023.e13250 (PMC9925976; doi:10.1016/j.heliyon.2023.e13250)
Supplement: Multimedia component 1 [file mmc1.docx]

**Proposed new authorship**

|  | First name | Family name | Affiliated institute | Email address |
| --- | --- | --- | --- | --- |
| 1st author | Amir Hossein | Khoshakhlagh | Department of Occupational Health, School of Health Kashan University of Medical Sciences, Kashan, Iran | [ah.khoshakhlagh@gmail.com](mailto:ah.khoshakhlagh@gmail.com) |
| 2st author | Saleh | Al Sulaie | Department of Industrial Engineering, College of Engineering in Al-Qunfudah, Umm Al-Qura University, Makkah 21955, Saudi Arabia | [smsulaie@uqu.edu.sa](mailto:smsulaie@uqu.edu.sa) |
| 3st author | Saeid | Yazdanirad (CA) | Social Determinants of Health Research Center, Shahrekord University of Medical Sciences, Shahrekord, Iran.  School of Health, Shahrekord University of Medical Sciences, Shahrekord, Iran. | saeedyazdanirad@gmail.com |
| 4st author | Robin | Marc Orr | Tactical Research Unit, Bond University, Gold Coast, Australia. | rorr@bond.edu.au |
| 5st author | Hossein | Dehdarirad | Medical Library & Information Sciences, School of Allied Medical Sciences, Tehran University of Medical Sciences, Tehran, Iran. | [dehdari.hossein@gmail.com](mailto:dehdari.hossein@gmail.com) |
| 6st author | Alireza | Milajerdi | Research Center of Biochemistry and Nutrition in Metabolic Diseases, Institude for Basic Sciences, Kashan University of Medical Sciences, Kashan, Iran | [miljerdi.a@gmail.com](mailto:miljerdi.a@gmail.com) |

**CA: corresponding author**

**Declaration of agreement. All authors, unchanged, new and removed must sign this declaration**

|  | First name | Family name | Agreement | Signature | date |
| --- | --- | --- | --- | --- | --- |
| 1st author | Amir Hossein | Khoshakhlagh | I agree to the proposed new authorship | 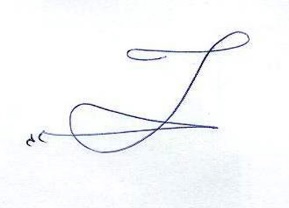 | 12 January 2023 |
| 2st author | Saleh | Al Sulaie | I agree to the proposed new authorship | 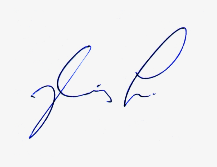 | 12 January 2023 |
| 3st author | Saeid | Yazdanirad (CA) | I agree to the proposed new authorship | 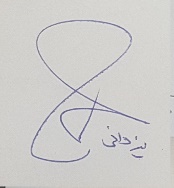 | 12 January 2023 |
| 4st author | Robin | Marc Orr | I agree to the proposed new authorship | 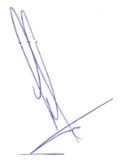 | 12 January 2023 |
| 5st author | Hossein | Dehdarirad | I agree to the proposed new authorship | 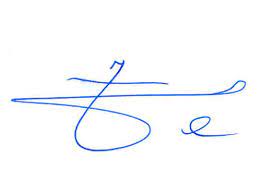 | 12 January 2023 |
| 6st author | Alireza | Milajerdi | I agree to the proposed new authorship | 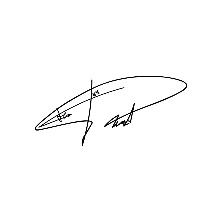 | 12 January 2023 |

**CA: corresponding author**

**New Author Contributions statement**

**Amir Hossein Khoshakhlagh:** conceived, designed, and carried out the study. Wrote the paper.

**Saleh Al Sulaie:** analyzed and interpreted the data. Wrote the paper.

**Saeid Yazdanirad:** conceived, designed, and carried out the study. Wrote the paper.

**Robin Marc Orr:** analyzed the data, interpreted the data, and drafted the manuscript. Wrote the paper.

**Hossein Dehdarirad:** analyzed and interpreted the data. Wrote the paper.

**Alireza Milajerdi:** analyzed and interpreted the data. Wrote the paper.

All the authors read and make the final corrections. The authors read and approved the final manuscript.
